# Development of modified rapid entire body assessment (MOREBA) method for predicting the risk of musculoskeletal disorders in the workplaces

**DOI:** 10.1186/s12891-022-05011-7

**Published:** 2022-01-24

**Authors:** Saeid Yazdanirad, Gholamhossein Pourtaghi, Mehdi Raei, Mohammad Ghasemi

**Affiliations:** Health Research Center, Lifestyle Institute, Baqiyatallah University of Medical Sciences, Tehran, Iran

**Keywords:** Prediction, Musculoskeletal disorders, Ergonomics, Risk assessment, Method, REBA

## Abstract

**Background:**

Rapid Entire Body Assessment (REBA) technique is one of the tools developed for predicting the risk of musculoskeletal disorders based on the effective risk factors. This method has several limitations. The present study was aimed to develop the Modified Rapid Entire Body Assessment (MOREBA) method to more accurately predict the risk of musculoskeletal disorders.

**Materials:**

This cross-sectional study was performed on 300 male workers of a steel factory with a variety of job tasks in Iran. Then, the information related to the various physical risk factors was extracted through observation of their duties and conversation with them. Also, the subjects were asked to complete the Persian version of Cornell musculoskeletal discomfort questionnaires (CMDQ). Then, a theoretical model was drawn in AMOS software. Computed coefficients were used to develop the MOREBA equation. In the end, the final scores were categorized by ROC curves, and the validation of the novel method was investigated using linear regression analysis.

**Results:**

The parameters evaluated in the MOREBA method included contact stress, rapid and sudden movement, throwing motion, hand-arm vibration, whole-body vibration, temperature, and work-rest cycle in addition to the parameters of the REBA method, including awkward posture, coupling, force, load, static activity, and repetitive activity. The results showed that the strain produced by the physical risk factors with the total effect coefficient of 0.783 could significantly affect the musculoskeletal symptoms. The computed coefficients of the risk factors were applied to develop a novel index. The final score of the MOREBA method was categorized into four levels by optimal cut-off points of 12.37, 16.51, and 24.35. Based on the results, the MOREBA and REBA methods could justify 67 and 55% of the variations of musculoskeletal symptoms, respectively.

**Conclusions:**

The results revealed that modifications conducted in the REBA method were effective, and the MOREBA method can provide a more accurate prediction of the risk of musculoskeletal disorders.

## Introduction

Musculoskeletal disorders (MSDs) are one of the major problems in various workplaces, such as office and operational environments. Musculoskeletal disorders are defined as a group of preventable disorders affecting nerves, tendons, muscles, and supporting structures such as intervertebral disks [1]. Some of these disorders include carpal tunnel syndrome, tendinitis, degenerative spine disease, thoracic outlet syndrome, and tension neck syndrome [2, 3]. The MSDs adversely impress on ability, life quality, and absence pattern in the workers [4]. It is estimated that 25% and 23% of European workers complain the back pain and muscle pain, respectively [5]. Based on the report of the Bureau of Labor Statistics in the United States, the MSDs represent 31.8% of all injuries and illnesses involving days away from work [6]. These statistics are worse in developing countries, such as Iran, because of old industries. The results of a review and meta-analysis study in Iran showed that the prevalence of work-related musculoskeletal disorders in the regions of neck, low back, upper back, shoulder, wrist, thigh, and knee were equal to 31.8%, 50%, 38.1%, 36.8%, 34.6%, 20.7%, and 42.1%, respectively [7]. Moreover, these disorders are increasing in various countries. Disability due to low back pain has been enhanced by more than 50% since 1990 [8]. Disability-adjusted life years (DALYs) related to musculoskeletal disorders have increased from 20.6 million to 30.9 million in 2010 [9]. MSDs have economic consequences in addition to health effects. It is estimated that the cost of treating the musculoskeletal injuries is equal to $127.4 billion in the United States [10]. The costs of work-related musculoskeletal disorders in developed and developing countries are variable from 0.5 to 2% of the GDP [11].

There are different factors affecting the occurrence of musculoskeletal disorders in the workplace. These factors can categorize into several groups, including physical, psychosocial, and personal determinants [12, 13]. Physical factors are an important group of effective factors. Herin et al. concluded that the factors of posture, heavy load, repetitive movement, vibration, and forceful effort can predict the chronic regional and multisite musculoskeletal pain in a working population [14]. Kumar et al., in a review study, identified the musculoskeletal risk factors of repetitive movement, awkward posture, work duration, load, force, and static muscle activity in cleaning occupation [15]. The results of a systematic review study performed by Seidel et al. showed that the physical risk factors of awkward posture, repetition, force, vibration, forceful grip, and combined exposure are associated with work-related musculoskeletal disorders [16]. Hildebrandt also stated that force exertions, dynamic loads, static loads, sudden and unexpected movements, repetitive loads, and environmental conditions are potentially hazardous workloads and working conditions [17]. Mitsuhiro et al. resulted that frequent throwing motion causes strain on the ulnar nerve in the regions of elbow and wrist [18]. The results of a study conducted by Pullopdissakul et al. indicated that high force, awkward posture, and contact stress are the main ergonomic factors associated with upper extremities musculoskeletal disorders [19]. Various tools have been developed for predicting the risk of musculoskeletal disorders based on the effective risk factors. Some of the observational methods include Rapid Upper Limb Assessment (RULA), Rapid Entire Body Assessment (REBA), Quick Exposure Check (QEC), Ovako Working Posture Analysis System (OWAS), and Novel Ergonomic Postural Assessment Method (NERPA). Among them, REBA is one of the known methods to evaluate different body parts, including upper limbs (arm, forearm, and wrist), lower extremities, trunk, and neck [20]. However, this tool has several limitations. REBA assesses only some of the mentioned risk factors, consisting of awkward posture, load/force, coupling, and repetitive and static activities [20]. Also, several assessments are required for different tasks of one person [21]. Moreover, REBA can overestimate the risk of MSDs in the employees. Sabini et al. observed that there is an overestimation of 45% in the cases assessed with REBA [22]. Therefore, this method can more accurately predict the risk of musculoskeletal disorders if these limitations are corrected. For this reason, the present study was aimed to develop the Modified Rapid Entire Body Assessment (MOREBA) method and evaluate its validation.

## Materials and methods

### Participants

This cross-sectional study was performed on 300 male workers of a steel factory with a variety of job tasks in Iran. For selecting the desired subjects, various parts of the stated industry including steelmaking, forging, machining, supporting, and administrative units carefully visited, and several occupations were chosen so that the extensive ranges of values related to the risk factors are collected. Then, 410 subjects employed in these job tasks were invited to the study and evaluated in terms of inclusion and exclusion criteria, of which 300 persons participated in the study. Data collected of these 300 subjects were used to develop and validate the novel method. Inclusion criteria included age range from 19 to 55 years, having work experience more than one year, not having a second job, and not having heavy physical activity in leisure time. Exclusion criteria also consisted of having a history of major trauma )such as driving, sports, and occupational accidents(, rheumatic diseases, spine surgeries, and large joint surgeries, and having musculoskeletal structural deformities )such as spinal abnormalities and genu varum(. Other exclusion criteria were long-term intake of corticosteroids and immunosuppressants and non-cooperation during the study.

### Sample size calculation

Given the aim of the present study, the minimum probable correlation between the score of the MOREBA and the score of musculoskeletal symptoms was considered by 0.2 and the sample size with a confidence level of 95% and a test power of 90% was computed as follow:

1 $$n=\frac{\left(Z_{1-{\displaystyle\frac a2}}+Z_{1-\beta}\right)}{w^2}+3\cong259$$

Where ${\text{Z}}_{1-\frac{{\upalpha }}{2}}$ is equal to 1.96 based on a confidence level of 95%, ${\text{Z}}_{1-{\upbeta }}$ is equal to 1.29 based on a test power of 90%, and W is equal to 0.203 based on a minimum correlation coefficient of 0.2. Therefore, the lowest sample size in this study was 259 persons.

### Data collection

 The protocol of this study was reviewed and approved by the Medical Ethics Committee of Baqiyatallah University of Medical Sciences (IR.BMSU.REC.1399.366). The demographic characteristics of the participants, including age, work experience, height, weight, and physical activity, were collected. Then, the information related to the risk factors of awkward posture, coupling, contact stress, static activity, repetitive activity, rapid and sudden movement, and throwing motion was recorded through observing the duties of the subjects and the data related to the risk factors of load, force, hand-arm vibration, whole-body vibration, temperature, and the work-rest cycle was gathered by an interview with them. Two persons of the researchers, who were well versed with the method, performed the assessments. Table 1 describes the guidance of scoring the factors. Based on this table, for evaluating the risk factors, the structured answers were used in steps of observation and interview. These risk factors were determined via a literature review in the valid databases and consultants with the specialists of occupational health and occupational medicine. The most worst and frequent positions related to each of body organs during work time were evaluated, and final scores related to each group (A and B) were estimated by tables of the REBA method [23]. The scores related to other factors were obtained based on the five-point Likert scales from 0 to 4 during work time. The final score related to force was calculated by scores of maximum load weight and load carrying time and the final score related to force was computed by scores of maximum force value and work time. Moreover, the most worst and frequent posture of each person was evaluated by the REBA method, and the final score was calculated using the assessment of factors of awkward posture, coupling, force/load, and muscle use [24]. Also, the subjects were asked to complete the Persian version of the Cornell musculoskeletal discomfort questionnaire (CMDQ) developed by Hedge et al. during rest periods. This questionnaire with a body map evaluates the frequency (never, 1-2 times last week, 3-4 times last week, once every day, and several times every day), intensity (slightly uncomfortable, moderately uncomfortable, and very uncomfortable), and interference (not at all, slightly interfered, and Substantially interfered) related to musculoskeletal symptoms in 12 regions of the human body at last week [25]. To calculate the total score of discomfort caused by musculoskeletal disorders, scores of frequency (0, 1.5, 3.5, 5, and 10), severity (1, 2, and 3), and interference (1, 2, and 3) at each of body regions was multiplied by each other and then obtained values were summed together [26]. The validity and reliability of the Persian version of this questionnaire were investigated by Afifzadeh et al. and confirmed by them in a steel factory. In this study, Cronbach’s alpha coefficient of frequency, intensity, and interference dimensions were computed by 0.955, 0.961, and 0.969 [27]. Finally, The accuracy of the answers in the completed questionnaires was checked by reviewing medical records and performing medical examinations.

### Predictive model

Structural equation modeling (SEM) was applied to predict the occurrence of musculoskeletal symptoms in the present study. For this purpose, a theoretical model was drawn, in which the total score of evaluated musculoskeletal discomforts was considered as a criterion variable, and the effect of strain resulting from the defined risk factors on it was investigated. Minimum acceptable factor loading was equal to 0.3 for retaining the item in the model [28]. The values of the Cronbach’s alpha coefficient, average variance extracted (AVE), and composite Reliability were also estimated. The minimum acceptable limits of these values were 0.7, 0.5, and 0.7, respectively [29, 30]. Finally, the indirect effect coefficients of the risk factors were extracted from the model and applied to develop the MOREBA equation. For calculating the indirect effect coefficient of each item, its direct effect coefficient was multiplied by the effect coefficient of the strain on musculoskeletal symptoms.

### Statistical analyses

Data were recorded into the statistical package for the social sciences (SPSS) version 18. For handling the missing data, the method of the mean of nearby points was applied to impute the missing data. The values out of the logical range related to the variables (outliers) were identified and corrected. The normality of variables was examined using Skew and kurtosis curves. Given that the statistical distribution of all variables was normal, the Pearson test was used to calculate the correlation coefficients between them. Then, a theoretical model was drawn in AMOS software. The fitness of this model was evaluated using absolute, comparative, and normed fit indices. After that, the MOREBA equation was written by indirect effect coefficients of the risk factors on the occurrence of musculoskeletal symptoms. Finally, the MOREBA score was categorized into four levels using receiver operator curves (ROC) analysis. The total scores of 450, 900, and 1350 related to the musculoskeletal discomforts were considered as boundaries of risk levels [31]. Nearest points to the ideal state in ROC curves were adopted as optimal cut-off points in the MOREBA index. The validity of the developed method was also examined by linear regression analysis. Moreover, the frequency distributions of the risk levels estimated by CMDQ, REBA, and MOREBA were computed.

**Table 1:** The guidance of scoring the factors

Factor		Scoring	Factor	Scoring
Posture group A		Assessing the most worst and frequent positions related to neck, trunk, and legs during work time and calculating score A using the table of REBA method.	Static activity	- Never (0) - Little (1) - Sometimes (2) - Much (3) - Very much (4)
Posture group B		Assessing the most worst and frequent positions related to upper arm, lower arm, and wrist during work time and calculating score B using the table of REBA method.	Repetitive activity	- Never (0) - Little (1) - Sometimes (2) - Much (3) - Very much (4)
Coupling status		- Very good (0) - Good (1) - Acceptable (2) - Poor (3) - Very poor (4)	Rapid and sudden movement	- Never (0) - Little (1) - Sometimes (2) - Much (3) - Very much (4)
Contact stress		- Never (0) - Little (1) - Sometimes (2) - Much (3) - Very much (4)	Throwing motion (such as hitting with a hammer or hand)	- Never (0) - Little (1) - Sometimes (2) - Much (3) - Very much (4)
Load	Maximum load weight	- Less than 5 kg (0) - 5 to 10 kg (1) - 10 to 15 kg (2) - 15 to 20 kg (3) - More than 20 kg (4)	Hand-arm vibration	- Never (0) - Little (1) - Sometimes (2) - Much (3) - Very much (4)
	Load-carrying time	- Never (0) - Less than 2 h (1) - 2 to 4 h (2) - 4 to 6 h (3) - More than 6 h (4)	Whole-body vibration	- Never (0) - Little (1) - Sometimes (2) - Much (3) - Very much (4)
Force	Maximum force value	- Less than 1 kg (0) - 1 to 2 kg (1) - 2 to 4 kg (2) - 4 to 6 kg (3) - More than 6 kg (4)	Air temperature	- Neutral (0) - Slightly warm or cool (1) - Warm or cool (2) - Hot or cold (3) - Very hot or very cold (4)
	Work time	- Less than 2 h (0) - 2 to 4 h (1) - 4 to 6 h (2) - 4 to 8 h (3) - More than 8 h (4)	Work – rest cycle (rest duration per two hours)	- Without rest (4) - 15 min (3) - 30 min (2) - 45 min (1) - 60 min and more (0)

## Results

Table 2 represents the statistical distribution of demographic characteristics and studied variables in the participants. The results of examining the Skew and kurtosis curves showed that the statistical distribution of all variables was normal. The CMDQ score related to musculoskeletal symptoms was variable from 0 to 1674. Table 3 reports the correlation matrix of the studied variables. The results indicated that there were meaningful correlations between all studied risk factors and CMDQ score (P<0.01). The highest correlation coefficients were related to the variables of posture group A (0.761) and group B (0.709), respectively.

**Table 2:** Statistical distribution of demographics characteristics and studied variables in the participants

Variable		Range	Mean	Standard deviation
Demographic parameters	Age (years)	20 – 56	38.21	10.17
	Weight (kilogram)	51.00 – 112.00	79.44	10.74
	Work experience (year)	1 – 34	15.86	9.39
	Height (meter)	1.55 – 1.93	1.76	0.06
	Body mass index (kilogram per square meter)	16.98 – 34.72	25.27	3.36
	Physical activity (hours per week)	0 – 20	2.85	2.29
Occupational parameters	Posture group A	1 - 9	4.76	1.93
	Posture group B	1 - 8	4.66	2.02
	Coupling	0 - 4	1.29	1.28
	Contact stress	0 – 4	0.52	0.28
	Load	0 – 4	0.94	0.69
	Force	0 – 4	1.27	1.20
	Static activity	0 – 4	2.29	1.06
	Repetitive activity	0 – 4	2.09	1.19
	Rapid movement	0 – 4	1.24	1.21
	Throwing motion	0 – 4	1.14	0.98
	Hand – arm vibration	0 – 4	1.14	0.65
	Whole body vibration	0 – 4	1.01	0.47
	Air temperature	0 – 4	1.24	1.15
	Work – rest cycle	0 – 4	2.78	0.77
CMDQ score		0 - 1674	536.82	511.51

**Table 3:** Correlation matrix of the studied variables

Variable		1	2	3	4	5	6	7	8	9	10	11	12	13	14	15
1	Posture group A	-														
2	Posture group B	0.843^**^	-													
3	Coupling	0.698^**^	0.734^**^	-												
4	Contact stress	0.327^**^	0.301^**^	0.119^*^	-											
5	Load	0.760^**^	0.793^**^	0.590^**^	0.315^**^	-										
6	Force	0.746^**^	0.765^**^	0.583^**^	0.281^**^	0.695^**^	-									
7	Static activity	0.653^**^	0.705^**^	0.523^**^	0.364^**^	0.646^**^	0.600^**^	-								
8	Repetitive activity	0.687^**^	0.727^**^	0.512^**^	0.361^**^	0.589^**^	0.606^**^	0.581^**^	-							
9	Rapid movement	0.610^**^	0.635^**^	0.450^**^	0.224^**^	0.580^**^	0.536^**^	0.517^**^	0.491^**^	-						
10	Throwing motion	0.703^**^	0.692^**^	0.506^**^	0.290^**^	0.673^**^	0.633^**^	0.587^**^	0.531^**^	0.483^**^	-					
11	Hand – arm vibration	0.397^**^	0.404^**^	0.325^**^	0.143^*^	0.383^**^	0.387^**^	0.353^**^	0.325^**^	0.291^**^	0.280^**^	-				
12	Whole body vibration	0.260^**^	0.357^**^	0.257^**^	0.076	0.298^**^	0.260^**^	0.191^**^	0.184^**^	0.165^**^	0.273^**^	0.192^**^	-			
13	Air temperature	0.394^**^	0.394^**^	0.368^**^	0.103	0.383^**^	0.319^**^	0.343^**^	0.281^**^	0.297^**^	0.316^**^	0.333^**^	0.173^**^	-		
14	Work – rest cycle	0.550^**^	0.572^**^	0.440^**^	0.203^**^	0.501^**^	0.534^**^	0.442^**^	0.526^**^	0.438^**^	0.458^**^	0.504^**^	0.175^**^	0.333^**^	-	
15	CMDQ score	0.761^**^	0.709^**^	0.600^**^	0.253^**^	0.634^**^	0.621^**^	0.593^**^	0.545^**^	0.537^**^	0.560^**^	0.389^**^	0.250^**^	0.450^**^	0.443^**^	-

^**^ P < 0.01

^*^ P < 0.05

**Fig. 1:**
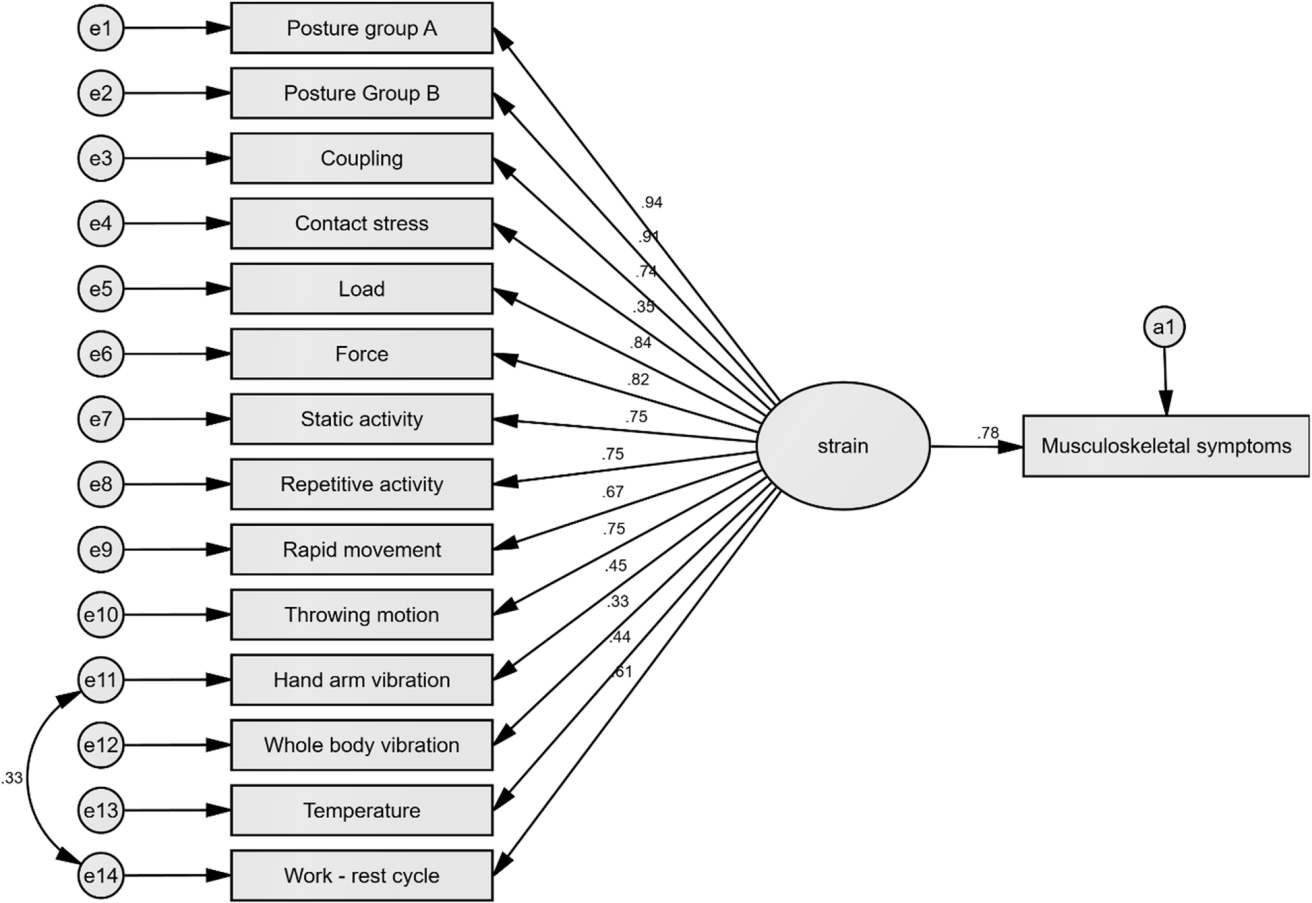
The theoretical model for predicting the occurrence of musculoskeletal symptoms due to occupational conditions

Figure 1 depicts the theoretical model for predicting the occurrence of musculoskeletal symptoms due to occupational conditions. The results showed that the strain produced by the risk factors with the total effect coefficient of 0.783 could significantly affect the musculoskeletal symptoms. Table 4 describes the effect coefficients of the risk factors on the variations of musculoskeletal symptoms. The factor loading (direct effect coefficient) of any item was not less than 0.3. Therefore, all items retain in the model. Posture group A (0.734) and B (0.714) had the greatest indirect effect coefficients on musculoskeletal symptoms, respectively. The lowest indirect coefficients also were related to variables of whole-body vibration (0.257) and contact stress (0.272), respectively. Table 5 reports the goodness-of-fit indices of the drawn model. Based on the results, the goodness-of-fit of this model was confirmed. The values of the average variance extracted (AVE), Cronbach’s alpha coefficient, and composite Reliability were computed by 0.50, 0.93, and 0.91, respectively. The results showed that these values were higher than the minimum acceptable limits.

**Table 4:** Effect coefficients of the variables in producing musculoskeletal symptoms

Variable	Direct effect	Indirect effect	P value
Posture group A	0.937	0.734	P < 0.001
Posture group B	0.912	0.714	P < 0.001
Coupling	0.743	0.582	P < 0.001
Contact stress	0.348	0.272	P < 0.001
Load	0.840	0.658	P < 0.001
Force	0.815	0.638	P < 0.001
Static activity	0.747	0.585	P < 0.001
Repetitive activity	0.748	0.586	P < 0.001
Rapid movement	0.670	0.525	P < 0.001
Throwing motion	0.751	0.588	P < 0.001
Hand – arm vibration	0.446	0.349	P < 0.001
Whole body vibration	0.328	0.257	P < 0.001
Air temperature	0.442	0.346	P < 0.001
Work – rest cycle	0.614	0.481	P < 0.001
Strain	0.783	-	P < 0.001

**Table 5:** The goodness-of-fit indices of the drawn model

index	Name	Threshold of Fitness	Obtained value
Absolute fitness indices	Goodness-of-fit index (GFI)	> 0.9	0.945
	Adjusted goodness-of-fit index (AGFI)	> 0.9	0.918
Comparative fitness indices	Normed fit index (NFI)	> 0.9	0.939
	Comparative fit index (CFI)	> 0.9	0.968
	Incremental fit index (IFI)	0-1	0.969
Normed fit index	Root mean squared error of approximation (RMSEA)	< 0.1	0.057
	Normed Chi-square (X^2^/df)	1-3	1.985

The indirect effect coefficients of the risk factors were used to develop the equation of Modified Rapid Entire Body Assessment (MOREBA) as follow:

2 $$MOREBAscore=\lbrack(0.734\times P_A)+(0.714\times P_B)+(0.582\times C)+(0.272\times CS)+(0.658\times L)+(0.638\times F)+(0.585\times SA)+(0.586\times RA)+(0.525\times RM)+(0.588\times TM)+(0.349\times HAV)+(0.257\times WBV)+(0.346\times T)+(0.481\times WRC)\rbrack$$

Where ${P}_{A}$ is the score of posture group A, ${P}_{B}$ is the score of posture group B, C is the score of coupling, CS is the score of contact stress, L is the score of load, F is score of force, SA is the score of static activity, RA is the score of repetitive activity, RM is the score of rapid movements, TM is the score of throwing motions, HAV is the score of hand-arm vibration, WBV is the score of whole-body vibration, T is the score of air temperature, and WRC is the score of work-rest cycle.

The scores of load and force parameters also are calculated as follow:

3 $$L=\frac{({t}_{L}\times {W}_{L})}{4}$$

Where ${t}_{L}$ is the score of load-carrying time and ${W}_{L}$ is the score of maximum load weight.

4 $$F=\frac{({t}_{w}\times {V}_{F})}{4}$$

Where ${t}_{w}$ is the score of work time and ${V}_{F}$ is the score of maximum force value.

**Fig. 2:**
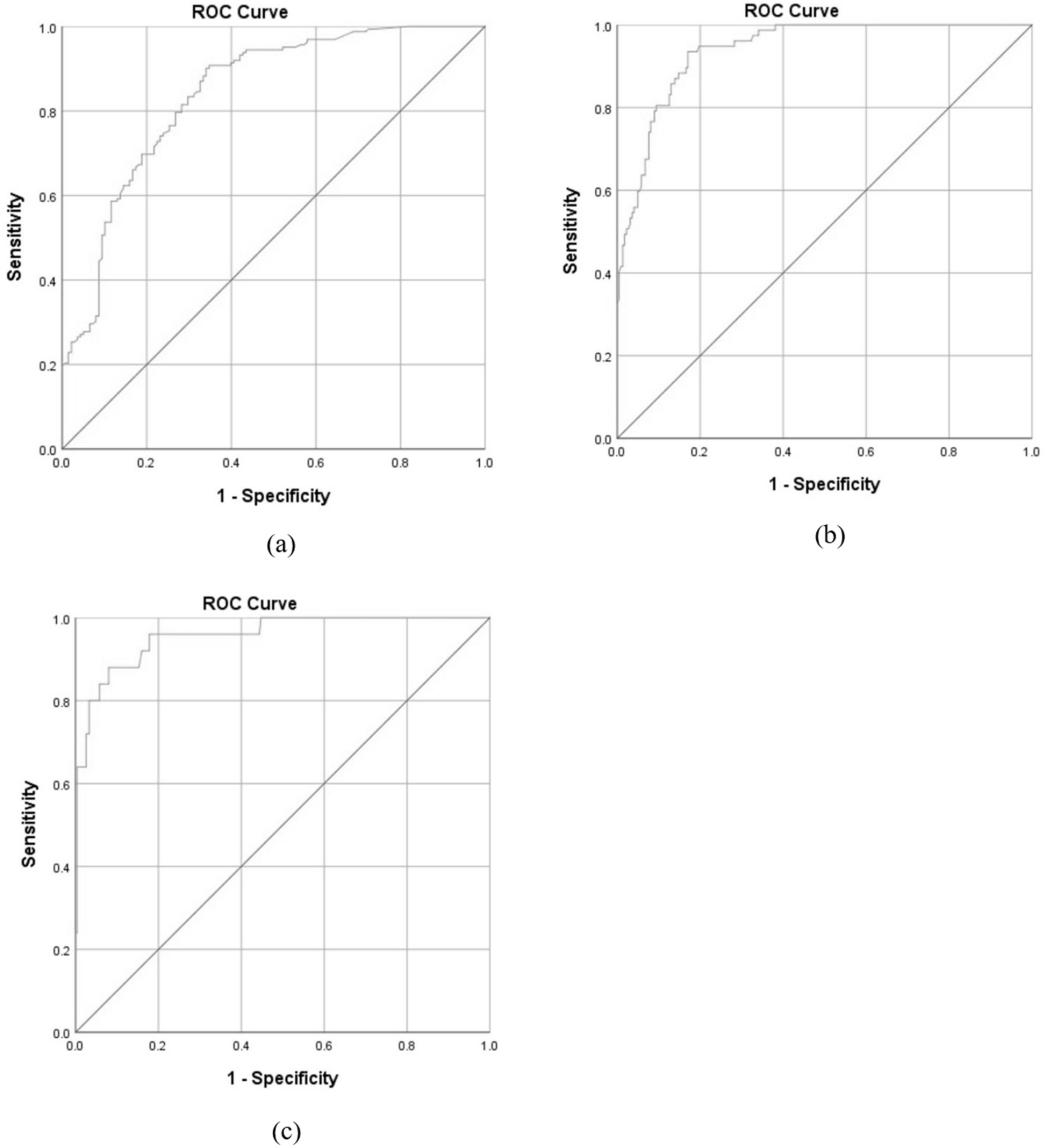
Receiver operating characteristic (ROC) curves of (**a**) low and moderate, (**b**) moderate and high, and (**c**) high and very high-risk zones

**Fig. 3:**
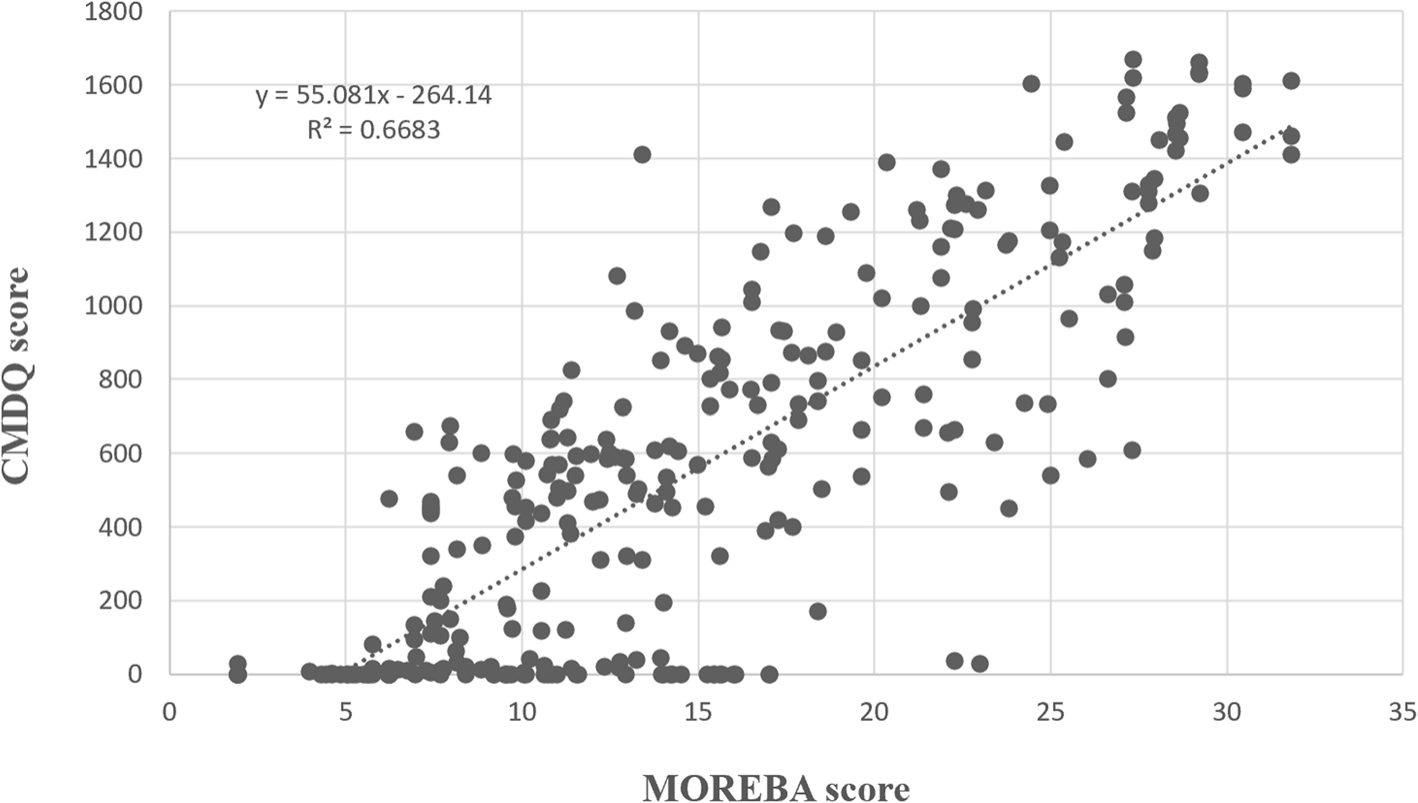
Linear regression curve between CMDQ score and MOREBA scores

**Fig. 4:**
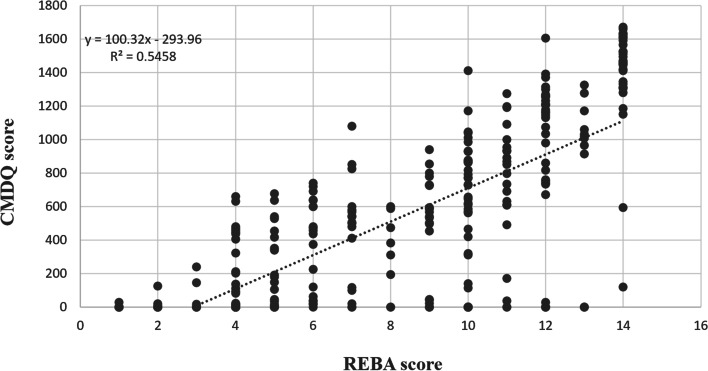
Linear regression curve between CMDQ score and REBA scores

**Fig. 5:**
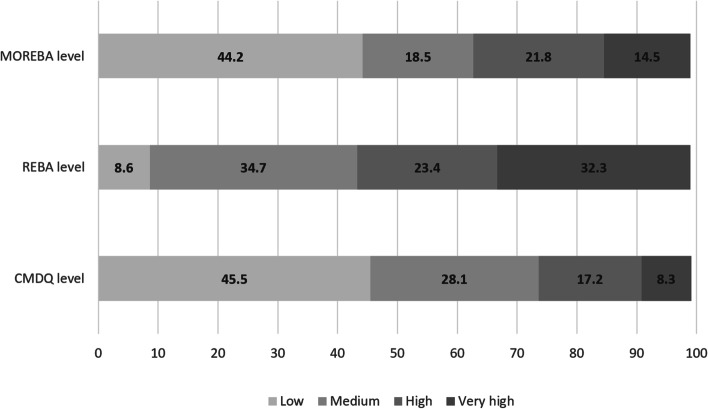
Frequency distribution of the risk levels estimated by CMDQ, REBA, and MOREBA

Figure 2 shows the receiver operating characteristic (ROC) curves. The results revealed that the optimal cut-off points between low and moderate risk, moderate and high risk, and high and very high risk included 12.37 (sensitivity = 0.796 and specificity = 0.732), 16.51 (sensitivity = 0.935 and specificity = 0.830), and 24.35 (sensitivity = 0.880 and specificity = 0.920), respectively. The area under of ROC curves (AUC) was also computed as 0.841 (95% CI: 0.0.796, 0.0.886) (p<0.001), 0.940 (95% CI: 0.915, 0.965) (p<0.001), and 0.957 (95% CI: 0.919, 0.996) (p<0.001), respectively. Table 6 represents the risk levels and equivalent MOREBA scores. Figures 3 and 4 show the linear regression curve between the CMDQ score and MOREBA score and REBA score, respectively. Based on the results, the MOREBA and REBA methods could justify 67 and 55% of the musculoskeletal symptoms, respectively. Moreover, Fig. 5 indicates the frequency distribution of the risk levels estimated by CMDQ, REBA, and MOREBA. As a result, the REBA method overestimates the risk level of musculoskeletal disorders while the relative frequency of different risk levels in MOREBA method was nearly close to those in CMDQ evaluation.

**Table 6:** The risk levels and equivalent MOREBA scores

Risk level	Equivalent score
Low	Less than 12.37
Moderate	12.37 to 16.50
High	16.51 to 24.35
Very high	More than 24.35

## Discussion

The results of the present study showed that the extensive range of values with normal distributions was collected. It indicates that a variety of situations have been studied, and the novel method can be used in workplaces with different physical risk factors. Based on the results, these various situations have been led to wide variations in the occurrence of musculoskeletal disorders. Moreover, it was observed that strain produced by the physical risk factors with the effect coefficient of 0.783 could significantly affect musculoskeletal disorders in people. Therefore, the MOREBA method can significantly predict the occurrence of musculoskeletal disorders. It may be due to the fact that almost all major physical risk factors were exploited in developing the novel method. The results of a study performed by Maakip et al. also showed that physical demands with 61% had the highest relative importance among various groups of effective factors for predicting musculoskeletal discomforts in Malaysian office workers [32]. However, the results of previous studies show that other groups of the risk factors such as individual and psychosocial items can impress on the occurrence of musculoskeletal disorders [32, 33]. So, if higher numbers of the risk factors are entered into the model, a more accurate prediction may be obtained.

Based on the results, the highest indirect effect coefficients on the occurrence of musculoskeletal symptoms were related to the factors of posture group A, posture group B, load, force, throwing motion, repetitive activity, static activity, and coupling, respectively. Most of these factors are essential items in other famous ergonomics risk assessment methods. In the methods of Rapid Upper Limb Assessment (RULA) and Novel Ergonomic Postural Assessment (NERPA), the factors of awkward posture, force/load, static activity, and repetitive activity are evaluated. The REBA method assesses the coupling in addition to factors of the RULA method [34]. In the occupational repetitive actions (OCRA) method, the factors of posture, force, frequency, recovery, duration, and some other items are examined [35]. In the Loading on the Upper Body Assessment (LUBA) technique, the body postures are appraised and other important factors are not considered [36]. In Job Strain Index (JSI), the factors of hand and wrist posture, force, duration, frequency, and work speed are evaluated [37]. Chander et al. also developed an observational method for Postural Ergonomic Risk Assessment (PERA) using three factors of posture, force, and work duration [38]. In the mentioned methods, posture and load/force variables have the most effect in calculating the final score of the risk assessment, respectively. Furthermore, Cheong et al. concluded that physical factors of posture and lifting/pulling had the greatest impact coefficients on work-related upper extremity musculoskeletal disorders in male workers [39]. the results of a study conducted by Mehralizadeh et al. indicated that the highest total effect coefficient on musculoskeletal complaints in the hospital nurses was related to the factor of body posture [40]. Therefore, the results of the present study are consistent with the results of previous studies. Moreover, the results showed that the factors of whole-body vibration, contact stress, temperature, and hand-arm vibration had the lowest indirect effect coefficients on the occurrence of musculoskeletal symptoms, respectively. It may be argued that the workers had less exposure to those compared to other risk factors in the workplaces and each of them affects fewer body regions. However, these factors also have significant effects on the occurrence of musculoskeletal disorders. Charles et al. concluded that occupational exposures to the whole body or hand-arm vibration resulted in musculoskeletal disorders in the shoulder and neck regions [41]. Based on the results of a study conducted by Pullopdissakul et al., factor of contact stress is associated with upper extremities musculoskeletal disorders [19]. Magnavita et al. found a significant interaction between temperature complaints and strain for upper limb disorders [42]. Therefore, the use of those in assessing the risk of musculoskeletal disorders can result in a more accurate prediction. However, a low number of ergonomic risk assessment methods, such as quick exposure check (QEC), has involved some of these factors.

The area under ROC (AUC) related to different risk levels was greater than 0.80 which indicates good diagnostic accuracy of curves [31]. Therefore, the scores can be easily and accurately interpreted based on this category. Moreover, the results of this study revealed that the MOREBA and REBA methods could justify 67 and 55% of the variations of musculoskeletal symptoms, respectively. It indicates that the prediction power of the MOREBA method compared to that of the REBA method has significantly improved. Also, it was found that the REBA method overestimates the risk level of musculoskeletal disorders while the relative frequency of different risk levels in MOREBA method was nearly close to those in CMDQ evaluation. The results of a study performed by Sabino et al. showed that there was an overestimation of 45% in the cases assessed by REBA [22]. Kee and Karwowski concluded that the REBA method overestimates some postures with low and moderate risk levels evaluated by the RULA method [43]. Moreover, in a study performed by Chiasson et al., REBA could not identify any low risk and it classified the majority of workstations (70%) in the high-risk category [44]. These findings are consistent with the results of the present study.

As one of the limitations in this study, all participants were male and the female workers were not studied. Moreover, objective examinations were not conducted for the diagnosis of musculoskeletal disorders. However, to ensure the correctness of the musculoskeletal discomforts reported by the participants in the CMDQ questionnaire, their medical records were reviewed and medical examinations were performed. It should be noted that the Iranian workers are annually examined by occupational medicine specialists in terms of various diseases, such as musculoskeletal disorders and their medical records are kept.

## Conclusions

The results demonstrated that modifications conducted in the REBA method were effective, and the novel method of MOREBA can provide a more accurate prediction of the risk levels of musculoskeletal disorders. The parameters of awkward posture, load, force, throwing motion, repetitive activity, static activity, and coupling in this method had the greatest effect coefficients, respectively. Moreover, the novel method has nearly rectified the problem of overestimating the risk of musculoskeletal disorders in the REBA method. Therefore, this method can be reliably used to predict the risk of musculoskeletal disorders produced by physical factors in the workplace. However, it is suggested that the MOREBA method be more validated by other assessors in other industries in future studies.

## Data Availability

All data analyzed during this step of study are included in this published article.

## Abbreviations

REBA: Rapid entire body assessment
MOREBA: modified rapid entire body assessment
CMDQ: Cornell musculoskeletal discomfort questionnaires
WMSDs: Work-related musculoskeletal disorders
DALYs: Disability-adjusted life years
RULA: Rapid upper limb assessment
QEC: Quick exposure check
OWAS: Ovako working posture analysis system
NERPA: Novel ergonomic postural assessment method
SEM: Structural equation modeling
ROC: Receiver operator curves
